# Toward Becoming an Accomplished Physician: Maimonides versus Galen

**DOI:** 10.5041/RMMJ.10060

**Published:** 2011-10-31

**Authors:** Samuel S. Kottek

**Affiliations:** History of Medicine, Hebrew University, Hadassah Medical School, Jerusalem, Israel

**Keywords:** Galen, Maimonides, medical education, perfection

## Abstract

Although Maimonides stated that perfection in the medical art, both in theoretical and in practical expertise, is very difficult to achieve, he did not accept Galen’s opinion, i.e. that perfection is beyond human capability. Any person seeking intellectual perfection should, according to Maimonides’ view, be fully trained in logic, in the natural sciences, and in theology. A physician is moreover requested to study and memorize basic medical literature; he must consider each patient as a sick individual, without neglecting the patient’s psychological disposition; and he should aim at inspiring confidence and trust, not only in his patient, but also in the latter’s environment. Even when feeling competent and trustworthy, the physician should not be conceited; here Maimonides insists on offering his personal experience, in a quite impressive way. This approach of Maimonides to the practice of medicine should be considered, even today, as a valuable incentive for patient-oriented medical education, as already expressed in the late eleventh century.

## INTRODUCTION

Maimonides (1137–1204) was not only a path-breaking theologian and philosopher, he was also a well trained physician; there is, however, no detailed curriculum of his medical studies. We only know that, while living in Fes, he received some medical instruction and was in contact with other physicians, as we will document further in this essay. Among the authors he cites in his ten medical works, the most often mentioned are Hippocrates, Galen, Avicenna, Rhazes, Avenzoar, and Averroes. We will, in this essay, focus on the Greco-Roman author Galen of Pergamon (second century CE)—who remained throughout the Middle Ages the most influential source in medical education. However, *Hippocrates*_(fifth century BCE) must be considered first.

The first aphorism of Hippocrates—“Life is short and the Art is long” (Gr. *makrós*)—is, no doubt, the most ominous statement of the “Father of Medicine.” The adjective *makrós* means indeed “long,” but also “abundant” or “far-reaching,” in other words, difficult to master.

In Maimonides’ commentary on this aphorism^1^ it says: “Perfection in this science, or rather art [i.e. medicine] takes longer to be mastered than human life [affords]. [However] this has been stated in order to warn candidate healers from easy-going studies.”

In order to document this warning, Maimonides delineates how the philosopher Al-Farabi divided the study of theoretical and practical medicine into seven parts. These include anatomy, hygiene, pathology, observation (i.e. examination), dietetics, follow-up of the healing process, and pharmacology (i.e. treatment). Maimonides adds terminology to these seven items, i.e. the names of diseases and drugs in various languages. We remember that he lived successively in Spain, in Morocco, and in Egypt; such migrations were then the lot of many physicians, Jewish ones in particular. Maimonides wrote a glossary of drugs,^2^ in which he used an impressive number of languages. It is well known that the same medicinal plant often appeared under different names, sometimes even in the same language. An accomplished physician was therefore supposed to be knowledgeable in these matters.

In his commentary on the first aphorism of Hippocrates, Maimonides issues a statement that may seem daring or even impossible to the modern reader. He contends that a student physician should memorize the huge corpus of theoretical and practical medicine. This does not only necessitate long and tedious work, it means that the physician will have to perform constant revision throughout his medical career.

The next stage in medical education is personal experience of treating the sick, which includes experience in diagnosis, in prognosis, and in the ways of prescribing drugs and adequate diet.

In one of his letters to a former student,^3^ Maimonides stated that in the evening, after a busy day, he reviewed the writings that dealt with the diseases he had treated during the day. Such a critical review and checking of one’s memory was definitely a requisite way in striving toward perfection. Incidentally, it is known that Maimonides regularly reviewed his theological writings, his *Mishneh Torah* in particular, till late in his life.

Let us now turn to *Galen* (131–201 CE). Most of Galen’s extensive works had been translated into Syriac and Arabic by Ḥunain ibn Yisḥaq and others; some of them only survived in their Arabic translation. A compendium of these writings, known as the *Sixteen Books of Galen*, was since Byzantine times in wide circulation.

In his commentary to Plato’s *Timaeus* (preserved in its Arabic translation only),^5^ Galen declares that it is beyond human beings’ capability to achieve perfection in the knowledge and in the expertise of the medical art. This statement will be adamantly opposed by Maimonides, as we shall see further.

In another work, admittedly not written by Galen himself, entitled *On Definitions*, we read: “The perfect [Gr. *téleios*] physician is one who has completed the whole scheme of theoretical and practical studies.” The author does not assert that perfection is beyond human reach. The next sentence reads: “The best [Gr. *aristos*; Lat. *optimus*] physician is the one who practices medicine according to the right doctrine [Gr. *orthon lógon*].” (On the Greek word *téleios*, and on *Ps.*-Galen’s *Definitiones Medicae*, see Reference 6.) There is, however, no detailed definition of “orthodox” medicine. This phrase intended most probably to exclude superstition, magic, and quackery.

Galen was, then and there, together with Avicenna and Rhazes, the main source of medical learning, for Maimonides as well as for his contemporaries. This does not mean that he always accepted Galen’s views without critical discussion.

## TOWARD PERFECTION

Before aiming at achieving perfection in medical practice, one should admittedly be, or become, an accomplished person.

We would like to quote from two non-medical texts of Maimonides, one philosophical—the *Guide of the Perplexed*—the other ethical—the *Eight Chapters.*

In the *Guide* (III, 54), Maimonides mentions four categories of perfection (Heb. *shelemut*). First mentioned is perfection in resources, second, perfection in health, third, in moral qualities, and, fourth, intellectual excellence. (Maimonides writes that perfection in property is of little essential value, although most human beings put it at the top of their endeavors. We remember the adage: “Who is rich?—One who is satisfied with what he owns.”^7^) These categories are cited in a sequence of growing importance. Regarding “perfection in resources,” this does not mean that one should become wealthy; however, one should be free from financial worries. Maimonides had indeed to cope with this problem: when his brother David, who provided for the financial needs of both families, suddenly perished at sea, Maimonides had to take over that responsibility. According to his own testimony, this caused him to be sick and depressed during a whole year, till he decided to become a practicing physician.

In the *Eight Chapters* (chapter IV),^8^ which are an introduction to his commentary on the *Fathers’ Aphorisms* (Heb. *Pirqei Avot*), Maimonides advocates adopting the medium line regarding the moral qualities. We quote (my own translation from the Hebrew):

Thus, the perfect man [Heb. *ha-adam ha-shalem*] should *constantly* call to mind his moral qualities [Heb. *midotav*], ponder his actions, and control his soul all day long. Each time he feels a propensity toward some extreme action, he should at once apply the accurate treatment in order to stop the progress of that tendency.

Maimonides adds that one should always keep in mind one’s moral weaknesses, and treat them in due time, for there is not anybody without shortcomings. In other words, no human being is essentially perfect, not even the biblical Moses; however, everyone should strive toward being perfect, while trying to control all his actions.

Returning to the *Guide* (I, 34), we shall now examine in what terms Rambam considers the difficulties that undermine philosophical accomplishment.

The reasons [for the difficulties] are that a person has, at the beginning [of his studies], very limited capabilities. A man does not own initially full mastership [Heb. *shelemuto ha-sofit*], although it exists in him virtually [Heb. *be-koaḥ*].

A lot of tenacity, of determination, and of work is required in order to become fully knowledgeable. In order to attain human perfection (Heb. *ha-shelemut ha-enoshit*), one has to master logic, the sciences that help in forming reflection, natural sciences (including medicine), and—ultimately—theology.

Further, in the same chapter, Maimonides adds that without first achieving excellent ethical behavior, one cannot attain accomplishment and perfection.

Earnest learning on the one side, ethical behavior on the other side, may lead to full accomplishment; however, very few are those who are able to reach this goal. It may be remarked that Rambam does not completely set aside full accomplishment in this context. Many human beings have virtually the possibility of becoming intellectually and ethically perfect, although very few achieve such ideal status.

## TOWARD PERFECTION IN MEDICINE

I would like to try and establish a tentative program of accomplished medical practice, according to Maimonides’ views featured in his medical works.

### Studying and Memorizing the Most Accurate Medical Works

In the *Book on Asthma*,^9^ chapter 13, Maimonides quotes an aphorism of Rhazes, in which he stresses how difficult it is to become a skilled physician. To which he adds:

The more accomplished one is in that science, the more precise his investigations are, the more doubts and difficult questions arise in him.

He will go into additional investigations and will hesitate in some of his answers.

Maimonides also remarks that even if understanding theoretical medicine from the literature may seem easy for someone who is in full possession of his faculties, the application of these notions to a practical case is often problematic, even for a trained and conscientious practitioner.^10^

As stated above, Maimonides described how hard and tiring his days of work were. Once his practicing was over, he reviewed and checked the difficult cases he had seen during the day, searching the literature that was at his disposal. He thus controlled his memory and checked himself constantly. This left him only the Sabbath for his theological studies, which were formerly his main field of interest.

### Discussing Difficult Cases with Colleagues

When Maimonides and his family lived in Fes, Morocco, he saw a patient who was “very strong;” however, after having undergone bleeding, the patient weakened and died the next night. Maimonides notes the following^11^: “A learned physician under whom I studied asked me: ‘Do you know the nature of the mistake this physician made in bleeding that patient?’” His teacher then explained that the patient was a glutton whose stomach (the cardia) had therefore been weakened. He should have known that Galen had forbidden bleeding in such cases, for it may cause fainting.^12^

From this story we learn two things: one, that Maimonides studied medicine in Fes; second, that he discussed practical cases with his teacher—he even quotes *in toto* the relevant passage from Galen. Both medical experience and remembrance of the adequate literature are thus documented.

Further in the same chapter, Maimonides describes another case, treated by four physicians, “all of them trained in this art.” The Sultan was prescribed theriac, but he died soon after ingestion. Maimonides inquired about the reason for death; he spoke to all the physicians involved, “in order to learn something useful from it,” but none of them answered him.^13^ Maimonides was in search of the *true* reason for the death, as formulated in the introduction to his commentary to the *Fathers’ Aphorisms* (*Pirqei Avot*): “Accept the truth from whatever source it comes.”

Maimonides moreover explained that his aim in recounting this case was to warn patients—not just physicians—to having recourse to strong drugs (such as theriac) only on the advice of an accomplished physician, and, even then, with great caution, only in case no other treatment may be devised.^14^

### Considering the Patient—Not Only the Disease

One of the central statements of Maimonides is the following:

One should never say: “This disease is similar to that [other] one.” … Nor should one say: “I have seen how my elders have treated [this disease] in such or such way.” [As a matter of fact] a physician does not treat a disease, he rather treats a sick person.^15^

To which he adds: “Every person who falls ill necessarily requires renewed consideration and reflection.”

Maimonides thus indicates that the constitution and the psychology of the patient must be taken into account. As stated in his *Regimen Sanitatis* (Heb. *Hanhagat Ha-Beriut*), Maimonides feels that a psychological assessment of the patient should even anticipate any medical intervention. “For every sick individual feels his/her heart constricted [Heb. *libo tsar*].”^16^

In other words, an accomplished physician should know how to adapt his way of addressing the patient according to the latter’s psychology.

Psychology was then a branch of Philosophy, and we thus understand better why even Galen said that a physician should be trained in Philosophy.

### Establishing Authority with the Patient and His Environment

In his *Commentary on Hippocrates’ Aphorisms*, Maimonides affirms that a physician who aims at doing his best for his patient’s benefit must have in view more than achieving an exact diagnosis and an adequate treatment for the disease. He must care for a full-fledged application of the treatment. Indeed, the patient might be reluctant to take a drug that is bitter or repulsive; and the care-takers might prefer taking advice from some popular quack or from a “wise woman.”^17^

The physician must therefore endeavor to gain full confidence from both patient and care-takers. Moreover, he should feel responsible for the removal of any impediment to the treatment; he should even help poor patients to purchase the drugs and/or to move to some healthier accommodation.

The duty to help poor patients applies to every individual, including physicians, but Maimonides feels a necessity to mention it here (cf. *Hilkhot ‛Aniyim* 10, 4–5).

According to Hippocrates, an effective way of gaining the patient’s trust is through accurate prognosis. We read:

I hold that it is an excellent thing for a physician to practice Prognosis. For if he discover and declare [*sic*], unaided, by the side of his patient, the past, and the future, and fill in the gaps in the account given by the sick, … men will confidently entrust themselves to him for treatment.^18^

In other words, the physician will thus show that he has full knowledge of the disease, even more than the patient is able to remember.

In this context, we can now better understand Maimonides’ statement in the *Book on Asthma*: “When the physician perfectly masters his art, then one will readily deliver his body and soul into his hands, and let him guide them according to his views.”^19^ This indeed conveys full confidence.

### Summary

According to Maimonides, a physician should, in order to attain perfection, or at least to strive at getting close to perfection, first master and memorize theoretical medicine; second, check carefully the relevant and trusted sources and/or discuss difficult cases with well trained colleagues; third, consider each patient individually, carefully weighing diagnosis, prognosis, and treatment; and fourth, gain full confidence of the patient and his environment.

We may argue that a number of these rules are quite relevant to actual medical education. They include patient-oriented medicine, fruitful collegial relationship, and continuous medical education.

## MAIMONIDES’ MODESTY

Maimonides was aware of the fact that his readers might consider that he regarded himself as the personification of a perfect physician. He therefore asserted:

Having heard my words, do not assume that I am the one into whose hands you should deliver your soul and body for treatment. May the Lord be my witness that I know for certain about myself that I too am among those who are deficient in this art, [who] stand in awe of it, and who find it difficult to achieve its goal.^20^

He adds that he does not state this out of modesty, or according to the ways of the pious (Heb. *Ḥassidim*), who maintain that their knowledge is deficient even when it is perfect. Maimonides apparently feared that his readers would suspect him of being conceited and would therefore not want to accept his advice.

In his ethical writings (cf. *Hilkhot De‛ot* I, 5 and II, 2), Maimonides explains how someone who has a tendency toward conceit should leave the ideal middle way of virtues and adopt extreme humility, at least for some time, until he feels that he may come back to the middle way. Modesty is the right way for a Sage.

## CONCLUSION

The ten medical works of Maimonides may seem like a drop in the sea of medieval medical literature. In comparison with Galen, Maimonides looks like a dwarf in front of a giant. Nevertheless, in the 25th chapter of his own *Aphorism*s (Heb. *Pirqei Moshé*), he enumerates a whole list of problematic statements of Galen, particularly on Philosophy. And he remarks:

[There is] a disease, which is so common that hardly one individual in a long period of time can avoid it. … This disease is that everyone imagines that he is more perfect than he really is; he wishes that all his opinions be considered perfectly true, [even when uttered] without toil or effort.^21^ (See Muntner’s Introduction in English, in particular “Maimonides against Galenus,” pp. XIX–XXXII.)

Galen himself—Maimonides says—suffered from that disease, not in his medical treatises, but in his philosophical and theological writings. We remember that for Galen perfection in medical practice was beyond human reach. Indeed, Maimonides asserts that he has not attained perfection, though refusing to accept the idea that perfection cannot be achieved. Did he say so out of humility, despite the fact that he openly denies it?

Let us try to define humility:

*Humility*: a state or quality of being humble in spirit; Freedom from pride and arrogance. (Compare to Webster’s definition, 22 and to a somewhat different approach to the topic of this essay in one of my studies 23 where I stressed particularly the psychological aspects, i.e. treatment of body and soul.)

In other words, humility is absence of pride based on one’s own achievements. I would like to argue that Maimonides would have accepted such a definition.

As he did in his other writings, philosophical or theological, Maimonides did his best in his medical works and in his practice, without a sense of pride. He therefore could not accept being presumed to be perfect. Even if he was considered as accomplished in the eyes of his patients, his ethical principles and way of life prevented him from such a belief; he was just *striving* toward perfection.

A real Sage is one who knows that he must sustain lifelong learning in order to uphold constant progress in knowledge.
